# Supplementary material for: Causal interplay between lactose intolerance and gut microbiota: a combined bidirectional Mendelian randomization and in vivo validation study
Source: Front Nutr. 2026 Jun 1;13:1803337. doi: 10.3389/fnut.2026.1803337 (PMC13265576; doi:10.3389/fnut.2026.1803337)
Supplement: Supplementary file 1 [file Data_Sheet_1.zip › supplementary materials/Forward/leave one out cross validation/ebi-a-GCST90027459.finngen_R12_E4_LACTONAS.pdf]

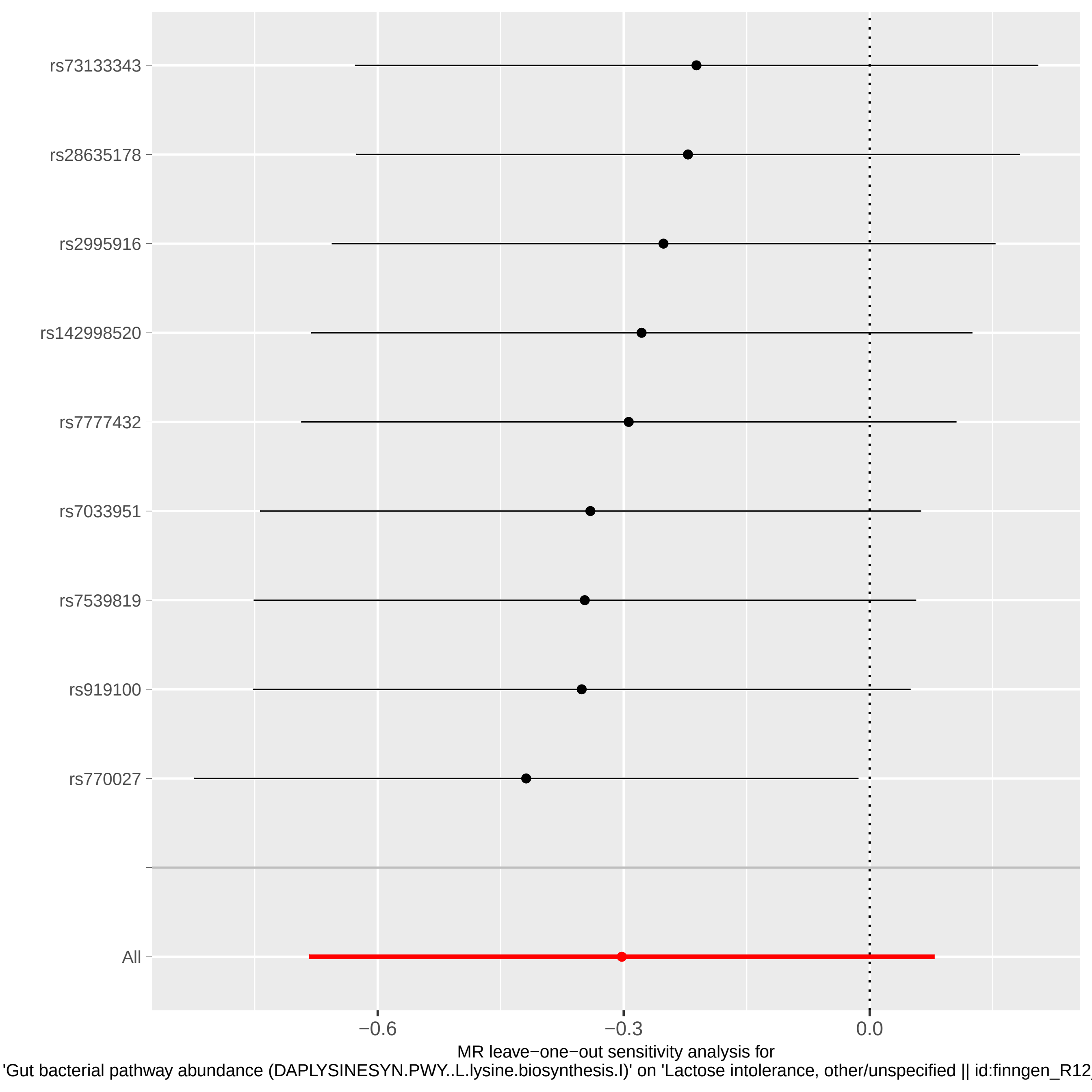
rs73133343
rs28635178
rs2995916
rs142998520
rs7777432
rs7033951
rs7539819
rs919100
rs770027
All
−0.6
−0.3
0.0
MR leave−one−out sensitivity analysis for
'Gut bacterial pathway abundance (DAPLYSINESYN.PWY..L.lysine.biosynthesis.I)' on 'Lactose intolerance, other/unspecified || id:finngen_R12
